# Changes in pharyngeal anatomy and apnea/hypopnea index after a mandibular advancement device

**DOI:** 10.5935/1984-0063.20210034

**Published:** 2022

**Authors:** Juan-Manuel Cortes-Mejia, Ana Boquete-Castro, Yoaly Arana-Lechuga, Guadalupe Jovanna Terán-Pérez, Katiuska Casarez-Cruz, Rosa Obdulia González-Robles, Javier Velázquez-Moctezuma

**Affiliations:** 1 Sleep Disorders Clinic, Universidad Autónoma Metropolitana, Sleep Disorder Clinic - Mexico City - Ciudad De Mexico - Mexico.; 2 Postgraduate in dentistry. Catholic University of San Antonio. Murcia, Spain.; 3 Neurosciences and Sleep Disorders Center., Sleep Disorder Clinic - Ciudad De Mexico - México.; 4 National Institute of Neurology and Neurosurgery, Imagen Center - Ciudad De Mexico - México.; 5 Universidad Autónoma Metropolitana, Mathematics Department - Ciudad de Mexico - Ciudad De Mexico.

**Keywords:** Primary Snoring, Sleep Apnea, sleep disorder, Mandibular Advance Device

## Abstract

**Objectives:**

This study aimed to evaluate the therapeutic effcacy of custom-made mandibular advancement devices (MAD) in the control of primary snoring and sleep apnea and to correlate with anatomical changes identified through imaging tests.

**Methods:**

Patients (n = 17) diagnosed with sleep apnea or primary snoring were included in this study and subsequently treated with MADs. Changes were assessed using a polysomnographic study (PSG), the Epworth Sleepiness Scale (ESS), and an imaging study with computed tomography scanning (CT). Studies were performed before and after the use of MAD. Anteroposterior measurements were taken in the sagittal plane at the hard palate, glottis, and supraglottic levels along the hard palate axis. Afterward, measurements were taken in the axial plane at the same levels along the hard palate axis.

**Results:**

From the six recorded measurements, the airway caliber increased by five. However, these changes were significant only in two measurements (sagittal hard palate and axial supraglottic). Snoring was controlled in 16 of the 17 subjects. From these sixteen, 12 subjects had a correct opening of the airway at the hard palate level. Moreover, daytime sleepiness decreased in all subjects.

**Discussion:**

Present results suggest that sagittal hard palate and axial supraglottic opening after use of MAD are mainly responsible for eliminating snoring and improve sleep apnea.

## INTRODUCTION

Obstructive sleep apnea (OSA) is a breathing disorder characterized by recurrent periods of upper airway obstruction during sleep. This obstruction can be complete (apneas) or partial (hypopneas). These recurrent events reduce oxygen saturation in the blood and cause brief arousals affecting sleep quality^1^. These pauses can be accompanied by snoring, which is defined as noisy breathing produced by oropharyngeal wall vibration^2^. Excessive daytime sleepiness (EDS) is the main symptom of patients with OSA and is present mainly when the patient is inactive^3,4^.

To assess the severity of the disorder, the apnea-hypopnea index (AHI) is commonly used. The number of apnea/hypopnea events per hour during sleep determines if OSA is mild, moderate, or severe^5^. When the only symptom is snoring, the diagnosis is primary snoring (PS).

Treatments for OSA range from behavioral measures, such as changing sleeping positions and avoiding muscle relaxers or central nervous system activators before sleep; to weight control, surgery, or non-invasive mechanical ventilation devices (continuous pressure airway positive - CPAP). In all cases, the rates of success depend on selecting the right treatment^6^.

Since the early 80s, MADs have been described in scientific literature as successful devices to control OSA in patients with mild to moderate OSA or who have PS due to tongue-based obstruction and soft palate hyperplasia^7^. MAD counteract the conditions that cause OSA by protruding the tongue base, or elevation of the tongue in case the patient is in a supine position, thus increasing the palatoglossal muscle tonus, expanding the pharynx, as well as normalizing and stabilizing the pharyngeal walls^8^.

The clinical practice guide of the American Sleep Disorders Academy and the American Academy of Dental Sleep Medicine indicates the MAD as the first choice in the treatment of PS. Also, the use of MAD in OSA is also recommended as the first alternative when the patient refuses the CPAP for any reason. When MAD is prescribed for an apneic patient, it is quite important that an experienced dentist led the procedure and that a personalized titration device is employed^9^.

The effectiveness of these appliances has been demonstrated using polysomnography (PSG) in various pre and post-treatment studies (with the MAD in situ)^10^. Computerized axial tomography and magnetic resonance imaging are objective tools that have been recently used to evaluate the change in pharyngeal dimensions with the device in situ^11,12^.

This study aimed to measure the airway caliber, using tomographic scanning, with and without a custom-made MAD. In addition, the degree of the upper airway opening was correlated with the improvement of the sleep breathing disorder.

## MATERIAL AND METHODS

Patients (17 adults: 39.8, ±10.7 years old; 9 males, 8 females) were recruited at the Sleep Disorders Center at UAM-I, after an open invitation through the social networks of the clinic. Volunteers older than 18 years that signed the informed consent were included. Those who required dental treatment were freely served. None of the volunteers reported previous use of MAD. Patients were diagnosed with PS or mild to moderate OSA. The patients were not overweight/obese (BMI *x*=24±1.9) having a neck circumference equal to or less than 40cm (Table 1).

**Table 1:** Demographic characteristics.

Variable	Mean (SD)
Age (years)	39.8 (10.7)
Gender	9 ♂ / 8**♀**
Body mass index	24 (1.9)
Neck circumference (cm)	39 (1.7)
Diagnosis	OSA (12) / PS (5)
Oxygen desaturation index	27 (34)

**Notes:** Diagnosis, OSA = Obstructive sleep apnea; PS = Primary snore.

Inclusion criteria were: diagnosis of PS; diagnosis of mild or moderate OSA; patients with severe OSA and intolerance to CPAP; base tongue obstruction; body mass index between 20 and 26; neck size until 41cm. Exclusion criteria were: somnolence as primary symptom; inappropriate oral hygiene or no teeth; severe gag reflex; disorders of the temporomandibular joint; psychiatric and/or neurological diseases; heart diseases or chronic obstructive pulmonary disease; an additional sleep disorder.

### Experimental design

The study was carried out following The Code of Ethics of the World Medical Association (declaration of Helsinki). The study was approved by the Academic Commission for Ethics in Biology and Health Sciences of the Metropolitan Autonomic University (UAM, Iztapalapa, Ciudad de Mexico) under the register number CECBS20-01. Informed consent was obtained for each participant in which the objective of the study was explained. This research was funded by CONACYT (National Council for Science and Technology, Mexico).

**Figure 1:**
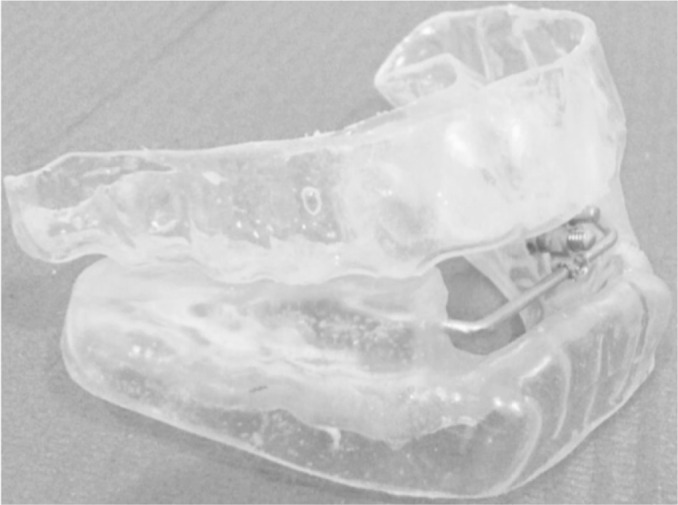
Picture of Mandibular Advance Device.

After a semi-structured interview in which the presence of apnea and snore was confirmed or rejected according to the bed companion; moreover, the patients were asked if they suffer from daytime sleepiness. After an all-night conventional PSG study, patients were diagnosed with PS or OSA. Snoring was recorded using a piezoelectric sensor attached to the anterior neck. To assess EDS, the ESS was applied, thereafter, a custom monobloc MAD (Figure 1), was made with resin for an average protrusion of 70%. Maximum protrusion and maximum retrusion were measured and 70% of advance was applied gradually to MAD from initial advance. All patients were instructed to use the MAD for 4 weeks before a second PSG study. Also, a CT scan was performed with and without the MAD to assess the airway opening.

### Polysomnographic studies

Nocturnal PSG studies were performed for 8 hours with no habituation period. A bipolar montage was used following the international 10/20 system of electrode placement in obtaining electrical brain activity. The studies included the analysis of electrooculogram (EOG), electromyogram (EMG), electrocardiogram (ECG), naso-oral flux (oronasal thermistor), thoracoabdominal movements, oxygen saturation, snoring, and body position. PSG studies were performed with Cadwell Easy, version 2 (Cadwell, Mexico). The polysomnographic data analysis also included an analysis of sleep architecture. The following elements were considered: sleep latency, rapid eye movement latency, the percentage of each sleep stage, wakefulness during the recordings, arousals, and awakenings in both studies.

### Titration protocol

The tolerance and attachment to the final MAD by using a training adaptive device was assessed. This allowed us to define the ideal level of advancement for each particular patient. According to the study of Wang et al. (2017)^13^, before using the definitive MAD, we performed all the titration process with a cheaper device manufactured with biocompatible materials of daily use in a dental office, inexpensive, custom and easy to handle. This provisional advice had an expansion screw located in the anterior zone. A dentist performed the advance (0.5mm) every weekly appointment. As well, the report of subjective response to snoring and OSA, including EDS were weekly obtained.

This provisional device was made with an acetate bilayer having one modified screw for expansion in the inter-arch space that allowed a millimetric advance that was gradually set observing the tolerance of the patient. Initially, around 50% of the maximum protrusion was registered with George Gauge. The maximum retrusion was considered when the lower incisors were located behind the upper incisors (negative value) and maximum protrusion was considered when the lower incisors were located in front of the upper incisors (positive value). Once the optimum advance was reached, the permanent device was designed, and the tomographic study was carried out.

As is well known, the mandibular advanced degree displays a positive correlation with MAD efficiency. According to Gauthier et al. (2009)^14^, the recommended mandibular advance varies between 50% and 75% taking into account the presence of temporomandibular articulation symptoms and the OSA degree. We established a 70% as a maximum advance as recommended, to avoid articular manifestations and patient withdrawal.

Concerning the rationale to use a monobloc, it must be mentioned that according to a report by Bloch et al. (2000)^15^, there were no significant differences when a monobloc and a two pieces device were successfully used in OSA. However, the authors report a clear preference of the patients for the monobloc. Also, the monobloc is cheaper and easier to manufacture.

### Tomographic scanning

**Figure 2:**
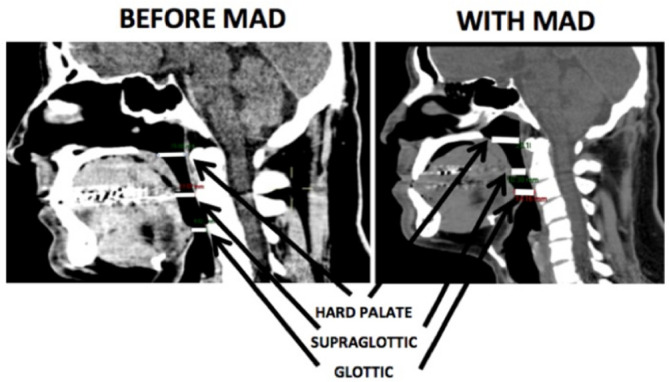
Sagittal section to show were the measurements were taken.

A 64-slice Siemens CT scanner was used for tomography scanning, with the patient in the supine position without using the MAD. A sagittal reconstruction was made and anteroposterior measurements were taken at the hard palate, supraglottic, and glottic levels along the hard palate axis. Then, axial plane measurements were taken at the same levels along the hard palate axis. Finally, the same procedure was performed while using MAD. Figure 2 depicts the sagittal images before and after MAD just to locate were the measurements were taken.

**Figure 3:**
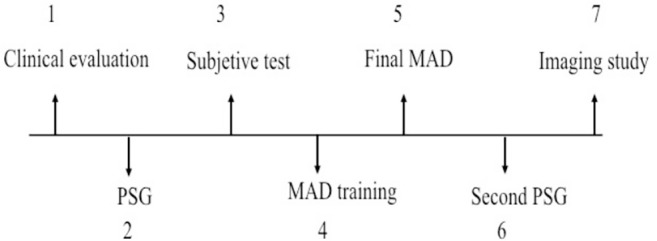
Timeline.

The study was performed according to the following timeline (Figure 3).

### Statistical analysis

Data were analyzed using NCSS and SPSS version 23. Shapiro-Wilk test was used to analyze distribution. When normality was found, a paired sample t-test was used. Otherwise, the Wilcoxon signed-rank test was used. The confidence level was set for *p*<0.05.

## RESULTS

Table 2 summarizes the results obtained concerning sleep patterns before and after treatment. As can be seen, percentage of sleep stages presented only a marginal change with a significant decrease in sleep latency after treatment. With this exemption, the remaining parameters showed no significant changes.

**Table 2:** Sleep variables. Median values of the variables related to sleep continuity and sleep-architecture before and after the use of MADs.

Variable	Before MAD Median (IQR)	After MAD Median (IQR)	*p*-value
Sleep latency (min.)	16 (66)	12 (47)	0.02*
Total time of sleep (min.)	424 (154)	437.5 (146.5)	0.25
Sleep efficiency (%)	88 (30.3)	89 (27.5)	0.92
WASO (min.)	26.6 (140)	30.2 (105)	0.82
Light sleep (%)	60.8 (34.6)	57.0 (28.7)	0.83
Slow wave sleep (%)	18.1(31.5)	20 (34.2)	0.14
REM sleep (%)	21.04 (12.3)	22.5 (14.6)	0.46
Arousal index	7.5 (12.3)	4.1 (17.1)	0.96

**Notes:** WASO = Wake after sleep onset; REM = Rapid eye movement; IQR = Interquartile range; Wilcoxon signed-rank test;

* *p*≤0.05.

**Figure 4:**
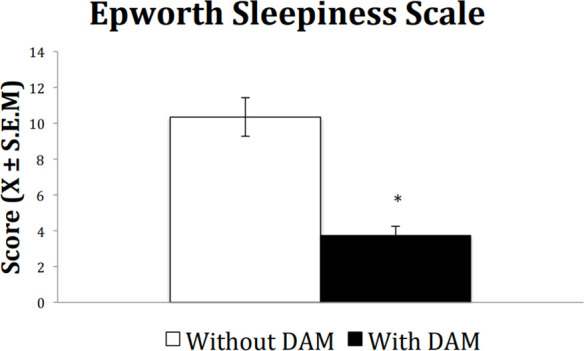
Excessive daytime sleepiness (Epworth Sleepiness Scale), pre (black) post (grey). Paired-Sample T-Test * p = 0.05.

Concerning EDS, results showed that after using the MAD the level decreased significantly from pathological to normal levels. Figure 4 shows ESS changes before and after treatment with MAD. Results shows that after treatment, the level of sleepiness decreased from an average of 10 to an average of less than 4, which lies within normal values.

Concerning PS and OSA symptomatology, most of the patients showed a significant improvement after the treatment. Figure 2 shows the apnea/hypopnea index and the snore index before and after MAD treatment. A very significant decrease is observed in both parameters. Both indexes reach normal values after the use of MAD. Concerning oximetric results, no significant differences were detected concerning the mean oxygen saturation. However, the minimal mean saturation showed significant differences (pre 81.5 (±7.3): post 86.1 (±2.4); *p*<0.05, Student t-test). As well, the desaturations index, which also shows significant differences (pre 13.6: post 2.9; *p*<0.05, Wilcoxon signed-rank test) (Table 3).

**Table 3:** Respiratory parameters.

n=17	Before MAD	After MAD	Test	*p*-value
Snore index (IQR)	174 (557.12)	8.4 (55)	Wilcoxon signed-rank test	0.00*
AHI (IQR)	9.9 (57)	1.3 (21.4)	Wilcoxon signed-rank test	0.00*
Mean SpO2% (SD)	90.6 (1.17)	90.8 (1.19)	Paired-sample t- test	0.075
Min SpO2% (SD)	81.5 (7.3)	86.1 (2.4)	Paired-sample t-test	0.01*
ODI (IQR)	13.6 (94)	2.9 (93.2)	Wilcoxon signed-rank test	0.03*

**Notes:**

* *p*<0.05; AHI = Apnea/hypopnea index; ODI: Oxygen desaturation index; IQR = Interquartile range; SD = Standard deviation.

As mentioned above, patients with PS (n=5) and patients with OSA (n=12; mild=5; moderate=4; severe=3), were included in the study. After treatment, PS patients decrease their snoring index by more than 50% and one decrease 30%. Concerning OSA patients, 10 (83%) decrease their AHI to normal values (<5), one decrease from severe to moderate, and one severe showed no beneficial changes.

**Figure 5:**
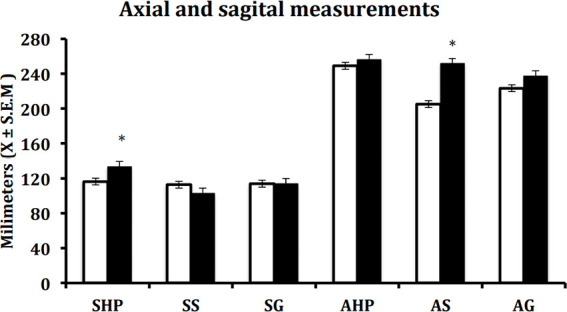
Axial and sagittal measurements of the upperway in hard palate, glottic, and supraglottic withouth (white bar) and whit MAD (black bar) MAD. The sagittal dimensions at the level of the hard palate and axial dimensions in the supraglottic area show a significant difference. Shapiro-Wilk. (SHP Sagittal Hard Palate, SS Sagittal Supraglottic, SG Sagittal Glottic AHP Axial Hard Palate, AS Axial Supraglottic, AG Axial Glottic). Paired-Sample T-Test * p = 0.05.

Regarding the morphological data, six measurements were taken before and after MAD treatment. As can be seen in Figure 5, statistically significant changes were observed only in the hard palate, both in the sagittal and in the axial planes.

**Figure 6:**
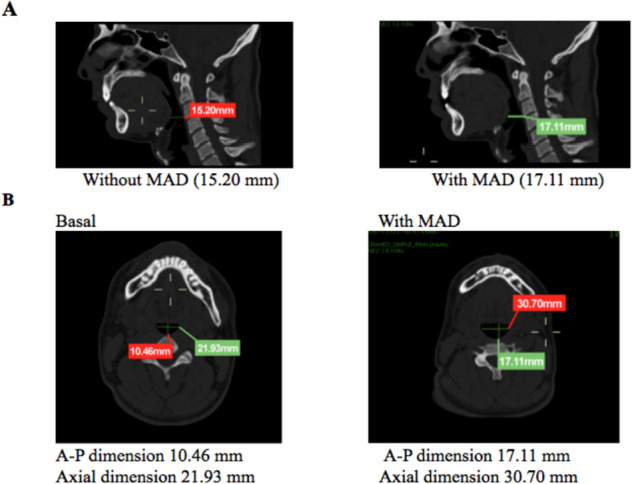
Example of the sagittal section and the difference in the pharyngeal dimension at the glottic level with and without the MAD. MAD (mandibular advancement device). Opening of the sagittal section at the glottic level (A: Pharyngeal dimensions without MAD; pharyngeal dimensions with MAD), anteroposterior (A-P) axial section, and the change in the pharyngeal dimension at the hard palate level (B), without and with MAD.

However, eight patients showed changes in the supraglottic and four in the supraglottic and hard palate. Concerning the sagittal plane, changes were observed in the pharyngeal dimension at the glottis while using the MAD. To clinical parameters, snoring was controlled in 16 of the 17 patients; of them, 12 showed statistically significant changes in the hard palate dimensions and 14 exhibited changes in the supraglottic dimensions. Three patients did not show changes in the hard palate. However, the supraglottic area did show a change and would explain the clinical improvement of the OSA (Figure 6).

Also, in a subanalysis of patients with and without OSA, results indicate that the five snoring patients showed significant differences after treatment (*p*<0.003, Wilcoxon signed-rank test). Concerning OSA patients, the AHI showed significant changes after treatment (*p*<0.001, Wilcoxon signed-rank test).

## DISCUSSION

Similar to previous reports, in this study, the effectiveness of MAD is corroborated resulting in a decrease in both the AHI and the subjective EDS values measured with the ESS^16^. It is known that MADs are a reliable and affordable option for controlling OSA^17^. Many studies have shown their effectiveness in reducing the presence of sleep apnea to normal levels comparing it with placebo, in patients with AHI<20^18^, and even long-term effectiveness^19^. However, other studies have not found significant results^20^.

Although CPAP is the first-choice treatment for OSA, it is not free of disadvantages. Besides its high cost, not all the patients reach full adherence. It has been reported that between 20 and 50% of patients cease the use of CPAP during the first year of treatment^21^. In addition, between 46 and 83% of the patients fail to use the CPAP more than 4 hours daily^22^. Furthermore, some sensitive patients can develop adverse collateral pathological manifestations^18^. Patients with severe OSA with no treatment due to CPAP intolerance, increase their mortality compared to a similar population treated with MAD^23^. Moreover, previous studies indicated that until 23% of patients with severe OSA and CPAP intolerance, display a significant improvement of OSA (less than 5/hr) after using a MAD^24,25^.

In the present study, 3 patients with severe OSA and CPAP intolerance were included. This decision was supported by the clinical practice guidelines of the American Academy of Sleep Medicine and the American Academy of Dental Sleep Medicine. In that report, the recommendation number 3 supports the notion that the use of MAD in severe OSA patients with CPAP intolerance is better than no treatment^9^. As mentioned above, two out of the three patients with severe OSA included in this study, showed beneficial changes using MAD.

Furthermore, according to a recent review by Wojda et al. (2019)^7^, to protrude mandible was therapeutically used in children with micrognathia since 1934^26^. Thereafter, several reports have communicated therapeutic effectiveness of several devices in OSA, regardless of different features such a size, material, and adaptation with the teeth. A milestone paper was reported in 1995, when the American Sleep Disorders Association published the clinical guidelines for the use of oral appliances in snoring and OSA^27^. This report supported the notion that oral appliances are a suitable treatment for PS and mild OSA. Since then, several reviews and studies have reported the benefits of oral appliances for snoring and OSA^28-30^.

Thus, the present results are following those reporting that oral appliances are a suitable treatment for snoring and mild OSA, decreasing EDS and increasing oxygen saturation. Moreover, image results in the present paper, indicate that two parameters of the respiratory upper way (sagittal hard palate and axial supraglottic) are the main features that account for snoring and apnea. However, image results in MAD and OSA are still scarce and controversial. In 2015, Geoghegan et al.^31^ reported anatomical changes using two different MADs and analyzing the results by lateral cephalometric radiographs; some anatomical changes were associated with the use of both MADs. However, Chen et al. (2019)^32^ reported no significant differences in the craniofacial anatomical structures analyzed by computed tomography scans. Recently, Mostafiz et al. (2019)^33^ using computed tomography and lateral cephalograms, reported a positive effect of MAD on maxillofacial disproportions but not in soft tissue obstruction. As well, they found significant differences using lateral cephalograms. Differences in the results of published papers could be due to methodological procedures in each case.

Johnston et al. (2002)^34^ described its effectiveness in reducing AHI and oxygen desaturation using MADs by comparing it with a placebo; however, these authors did not find significant differences in subjective ESS scales. It has been documented that the patients who responded less to the use of MADs were those with severe OSA before treatment^19^; studies like this have cause an increase in the recommendation for the use of MADs to be limited to those patients presenting snoring and moderate OSA but without EDS.

These differences could be explained because in the present study, unlike the aforementioned studies, patients with severe OSA index were excluded.

The fact that ESS scores decreased after treatment until the normal range, suggests that MADs are effective in controlling the daytime symptoms of OSA^17^. Since the first report by Johns et al., in 1991^35^, ESS has been widely used as a reliable method to assess sleepiness. The original paper suggests that a result of 10 or more could be considered pathological. According to a review by Johns et al., in 1998^36^, less than 10 points should be considered located in the normal range, while a value between 10 and 12 reflects mild somnolence and more than 12, excessive somnolence.

Moreover, in a study aimed to determine the reliability of ESS to detect somnolence in mild OSA, authors reported that sensitivity of the test increased from 66% to 76% when the cutting point was decreased from 10 to 8 to detect a patient with pathological somnolence^37^. Also, the scale has been validated for a great number of languages and commonly, a value of 10 or more is considered pathological (for review see: 7).

Another relevant variable involved in the adequate therapeutic response with the use of MADs is the upper airway opening achieved. Some researchers have shown a positive correlation between the opening of the pharynx with the use of MADs during wakefulness and the response to treatment, both in the decrease of the IAH index and in the sleepiness. This current study’s results are in accordance with other authors who have suggested that craniometrical parameters can be a good predictor of the adequate response of MADs, even in patients with obesity and severe OSA^31^.

It must be noticed that we obtained significant opening values in the upper airway after the use of MAD only in the sagittal hard palate and in the axial supraglottic. According to the results showing the decrease of snoring and apnea index, it seems that the changes in these parameters are enough to normalize both snoring and apnea index.

In past years, image diagnosis has been implemented successfully in medicine and dentistry, and more recently, even in dental sleep medicine, through bi-dimensional and three-dimensional analysis of the upper airway^38^. Retroglottic and retropalatal areas have been described as predictors of OSA^39^. Moreover, changes in the volume of the upper airway related to OSA have been determined using a cone beam computerized tomography (CBCT) scan^40^.

The present study assessed the digital image analysis of the upper airway as a complementary tool in the diagnosis of OSA. However, we acknowledge the need to incorporate dynamic assessment of the upper way during inspiration and expiration as well as volumetric measurements. The results obtained strongly suggest that future studies in this field, must be followed up by CBCT and routine PSG.

It must be mentioned that cephalometry is a common technique used for the assessment of respiratory changes after MAD. However, the images frequently display distortions^41^. On the other hand, CT allows the capture of sequential images during the respiratory cycle, whit lees radiation exposure compared to RMN^38^.

## CONCLUSION

From the present study, it can be concluded that the increase of the airway caliber is related to the reduction of snoring and the number of pauses in breathing, as well as with daytime sleepiness levels. This confirms the effectiveness of the MADs in the control of PS and mild to moderate OSA.

The use of a training device to gradually find the optimal advancement for a particular patient seems to ensure their willingness to go through with the treatment. Also, it seems that only two parameters of the respiratory upper way (sagittal hard palate and axial supraglottic) are responsible for the presence of PS and OSA. In the present study, the increase of these parameters results in the normalization of PS and OSA.

One major limitation of the present study is the size of the sample and new observations should be done to consolidate the present results. In addition, future studies should include volumetric studies and a questionnaire about snore. Further research is needed to fully elucidate the significance of these findings.
